# Post stroke health-related quality of life, stroke severity and function: A longitudinal cohort study

**DOI:** 10.4102/ajod.v11i0.947

**Published:** 2022-01-26

**Authors:** Tasneem Hartley, Marlette Burger, Gakeemah Inglis-Jassiem

**Affiliations:** 1 Division of Physiotherapy, Faculty of Medicine and Health Sciences, Stellenbosch University, Cape Town, South Africa; 2 Department of Physical Therapy and Rehabilitation Science, College of Health Sciences, Qatar University, Doha, Qatar

**Keywords:** stroke, cerebrovascular accident, health-related quality of life, function, South Africa

## Abstract

**Background:**

Health related quality of life (HRQoL) is a determinant of physical, social and emotional well-being post-stroke.

**Objectives:**

This study aimed to correlate self-reported HRQoL with activities of daily living (ADL) and stroke severity.

**Method:**

A longitudinal observational study was conducted at a rehabilitation centre in the Western Cape, South Africa. Stroke inpatients were sampled over 6 months. The Euro-QoL Five Dimensions instrument (EQ5D-3L) for self-reported HRQoL, Barthel Index (BI) for function and independence in ADL, and modified Rankin Scale (mRS) for stroke severity were administered on admission and discharge. Statistical analysis was performed using Statistical Package for the Social Sciences (SPSS) version 25.

**Results:**

Of the 54 potential participants, 49 met inclusion criteria and 41 completed reassessments (median age 48 years [interquartile range {IQR} 39–60]; median length of stay 53 days [IQR 46–60]). Most participants had infarctions (89.90%); with hypertension or diabetes risk factors (73.47% and 28.57%, respectively). The BI and mRS scores improved significantly (*p* < 0.001) with very strong correlation between scores (rs = -0.874, *p* < 0.001); indicating a trend of decreased stroke severity as function improved. The EQ5D Visual Analog Scale (VAS) scores (*p* < 0.001) and domains mobility, self-care, usual activities (*p* < 0.001) and pain/discomfort (*p* = 0.034) improved significantly. The anxiety/depression domain showed a non-significant change (*p* = 0.378). A weak negative significant correlation existed between EQ5D VAS and mRS scores (rs = -0.362; *p* = 0.02); indicating a trend that HRQoL was not improving to the degree stroke severity decreased. A weak positive significant correlation was seen between EQ5D VAS and BI scores (rs = 0.329; *p* = 0.036).

**Conclusion:**

Although an improvement was noted in HRQoL, EQ5D VAS scores tended not to improve as strongly, despite significant improvements in function and stroke severity. These findings demonstrate the need for psychological support and pain management interventions for adjustment post-stroke.

## Introduction

Globally, stroke is the third most common cause of disability and an increasing global burden (Bertram et al. 2008; Lozano et al. 2012). According to Naghavi et al. (2017), stroke and ischaemic heart disease accounted for 85.1% of cardiovascular disease-related deaths globally. Taylor and Ntusi (2019) stated that if the current trends in stroke continue, there would be 20 million annual stroke-related deaths and 70 million stroke survivors globally by 2030. It is well-documented that 80% of strokes occur in low- to middle-income countries (LMICs) (Maredza, Bertram & Tollman 2015; Pate et al. 1995; Taylor & Ntusi 2019). A review by Kengne and Anderson (2006) indicated that there is also an increased incidence of young stroke in LMICs, as compared to high-income countries. This phenomenon affects the Sub-Saharan African region in particular (Kengne & Anderson 2006). Furthermore, global trends highlight an increased rate of ischaemic stroke in young adults. This increase may be associated with infective diseases such as HIV (Boot et al. 2020). South Africa has one of the highest rates of HIV and AIDS infections, and young stroke within the country has been attributed to HIV and AIDS (Taylor & Ntusi 2019). As a result of the increase in these stroke risk factors, Taylor and Ntusi (2019) described an epidemiological transition in South Africa where stroke occurs in a relatively younger population.

With the high rate of non-communicable diseases (NCDs), lack of physical activity, poor diet and rate of alcohol consumption, stroke has become the eighth most common cause of years of life lost to illness and ninth cause of disability in South Africa (Bryer et al. 2010; Maredza et al. 2016). The potential long-lasting consequences of stroke-related disability could be minimised with effective and efficient stroke care services. Health related quality of life (HRQoL) is a determinant of physical, social and emotional well-being post-stroke (Hartigan et al. 2011; Hunger et al. 2012). Hence, understanding the subjective perception of an individual’s HRQoL with regards to recovery post-stroke may guide the planning and delivery of targeted and/or appropriate person-centred stroke rehabilitation services.

Stroke-related disability results in a great financial burden on stroke survivors and their families because of the stroke-related care requirements and the patients potentially not being able to return to productive activity (Langhorne, Bernhardt & Kwakkel 2011; Rhoda 2014). With the increase in stroke rate and the number of strokes occurring in LMICs, there would be a concomitant increase of stroke-related economic burden borne by stroke survivors in these countries too (Taylor & Ntusi 2019). Hence, understanding the perception of an individual’s HRQoL with regards to recovery post stroke is imperative in improving healthcare services which are better geared towards stroke recovery (Katona et al. 2015; Jalali & Dutta 2012; Tang et al. 2013). Stroke has varying degrees of severity which can affect mobility and independence in activities of daily living (ADL) which is strongly associated with HRQoL (Markus 2012). The HRQoL encompasses not only physical health status but psychological, social functions as well as environmental factors (Badaru et al. 2015). Thus, young stroke may have detrimental effects on quality of life at an age in which individuals are productive members of society (Hartley et al. 2020).

Recovery post stroke could take months to years, and is a formidable journey with many individuals not regaining their previous level of function (Yeoh et al. 2019). Rehabilitation services are centred around functional status with little emphasis on the patient’s perception of function and quality of life. A patient’s perception of their function and quality of life is essential in post-stroke recovery and a determinant for progress as physical, social and emotional well-being are inter-related (Duff 2012; Guise et al. 2010; Hartigan et al. 2011; Hunger et al. 2012; Ntsiea, Van Aswegen & Olorunju 2013). Previous cohort studies report that poor HRQoL is associated with depression, which is a common occurrence post stroke as 1 in 5 stroke patients experience anxiety and 1 in 3 experience depression (Ojagbemi et al. 2017a, 2017b; Unibaso-Markaida et al. 2019). Anxiety and depression within stroke patients are strongly associated with poorer functional outcomes, poorer cognitive impairment, and family support (Ojagbemi et al. 2017a). In addition, limited social interaction which is inter-related with cognitive and functional impairments post stroke, further negatively impacts perceptions of HRQoL (Isaac, Stewart & Krishnamoorthy 2010; Unibaso-Markaida et al. 2019). Previous studies report that greater stroke severity and poorer physical functioning adversely affected HRQoL (Badaru et al. 2015; Rhoda 2014). In addition, 29% – 85% of stroke patients experience anxious and/or depressive symptoms which have been shown to negatively influence physical functioning (Badaru et al. 2015). The physical consequences of stroke in addition to the psychological and emotional detriments decrease the social functioning of stroke survivors which all adversely affect their HRQoL (Badaru et al. 2015). Majority of stroke patients in South Africa, are unable to return to previous day to day activities and have increased levels of depression and anxiety, which in turn affect their overall HRQoL (Rhoda et al. 2014). Previous longitudinal studies report that HRQoL in stroke patients does improve with rehabilitation 6–12-months post stroke as compared to the acute phases (Lynch et al. 2020). However, when comparing the HRQoL of stroke patients to age matched healthy individuals, the HRQoL of stroke patients is poorer (Boot et al. 2020; Rhoda 2014).

Although many studies have reported on the HRQoL post stroke in South Africa, this is the first study to correlate HRQoL of stroke inpatients using the Euro-QoL Five Dimensions instrument (EQ5D) with stroke severity and independence in ADL. This tool has been validated and shown to be responsive in the stroke population (Dorman et al. 1997), and shows good accuracy when completed by a proxy (Pickard et al. 2004). In addition, it has been validated in the South African population (Jelsma & Ferguson 2004).

## Methods

We conducted a prospective longitudinal study with a pre- and post-design where patients were assessed upon admission and prior discharge from inpatient rehabilitation. The Stellenbosch University Health Research Ethics Committee (HREC) approved this study (S15/10/232) and all participants provided written informed consent. The study was conducted at an inpatient rehabilitation setting, the Western Cape Rehabilitation Centre (WCRC) based in Cape Town, South Africa. The WCRC has a catchment area that encompasses the entire Western Cape province as well as the surrounding provinces with some admissions from the Northern and Eastern Cape provinces. Some patients are also referred from neighbouring countries such as Lesotho, Zimbabwe and Namibia.

The data reported in this article pertains to HRQoL (EQ5D), stroke severity and functional status (Modified Rankin Scale [mRS] and Barthel Index [BI]) which formed part of a larger prospective longitudinal descriptive cohort study (Hartley et al. 2020). The rehabilitation centre provides high-intensity, specialised inpatient rehabilitation, which accepts appropriate referrals from all levels of healthcare services (i.e. tertiary, secondary, district and primary level healthcare services) in South Africa. The rehabilitation team may include doctors, physiotherapists, occupational therapists, speech therapists, dietetics, as well as appropriate referrals to social workers and psychologists where necessary. Each patient is holistically assessed on admission by the multidisciplinary team (MDT) and a tailored individualised rehabilitation programme is developed (WCRC 2021). These patient-centred interventions are tailored to each individual’s cognitive and physical abilities as well as mental health needs. Rehabilitation is scheduled Monday to Friday, with 30 min to hourly sessions per therapy session. Sessions with psychology and social work services may not be as frequent, but are provided to meet specific patients’ needs. The intensity and frequency of sessions would be determined by each patient’s capacity, endurance and participation in therapy along with available therapist resources. Regular reassessments and MDT meetings are scheduled throughout the rehabilitation process to monitor patient progress and make necessary adjustments to goals and interventions. The rehabilitation process includes family education and discharge planning. In addition, prior to discharge, patients may go home for weekend leave to facilitate integration of rehabilitation activities at home. Where possible, home visits are scheduled to assess the home environment and apply interventions appropriately and with feedback, therapists may adapt the home environment where necessary (Hartley [WCRC] pers. int., 25 August 2015).

An admissions clerk from the rehabilitation centre assisted with the recruitment process and was the liaison between rehabilitation clinicians and the primary investigator (PI). All stroke patients admitted to the rehabilitation centre were eligible for the study. Thus, a convenient sampling method was applied over a 6-month period where all eligible participants were screened for inclusion in the sample for this study. The clinicians were educated on the inclusion criteria and referred potential participants to the PI. The PI would gain consent from potential participants and verify if they met the inclusion criteria via perusing their medical records. Participants were included if they were 18 years and older, with a first ever stroke, and able to respond to verbal cues or commands in English and/or Afrikaans and/or isiXhosa (Hartley et al. 2020). There was no upper limit to age in terms of eligibility. Participants who did not meet the inclusion criteria or were diagnosed with expressive aphasia by their treating therapists were excluded. The stroke diagnosis in most cases were confirmed by CT-scan or MRI by the referring healthcare facility.

**FIGURE 1:**
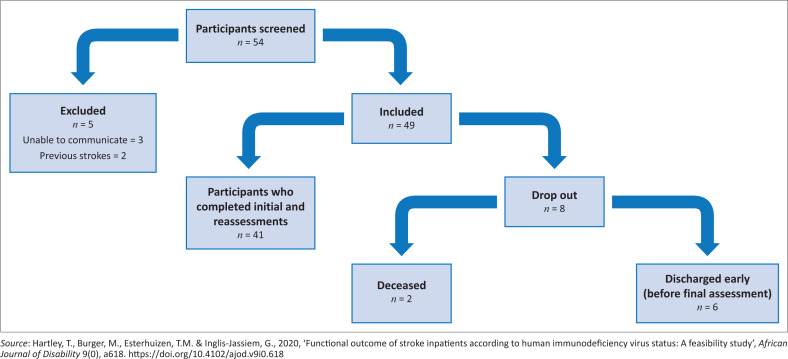
Recruitment flow chart.

Participants were assessed on admission and reassessed on discharge. Initially 54 potential participants were screened of which 49 met the inclusion criteria. There was a drop-out rate of eight participants on account of death unrelated to stroke (*n* = 2) and being discharged prior to reassessment (*n* = 6). This resulted in 41 participants completing reassessment prior to discharge (see Figure 1).

Outcome measures utilised on admission and prior to discharge included the EQ5D for HRQoL, the mRS for stroke severity and the BI for functional status. These measures were utilised on admission and prior to discharge by the PI. The EQ5D is a self-reported HRQoL measurement tool used in various conditions including stroke (Hunger et al. 2012). The EQ5D explores five domains namely mobility, self-care, usual activities, pain/discomfort, and anxiety/depression, which have three level descriptors, namely experiencing no problems, some problems or extreme problems within each domain. Level one is the more favourable option where participants experience no difficulty, nor any symptoms, whereas level three describes the inability to perform mobility, self-care and usual activities or experiencing extreme symptoms of pain/discomfort and anxiety/depression. A separate item within the EQ5D questionnaire is the VAS which required participants to score their health status using a rating, 0–100; the score 0 being worst and 100 the best imaginable health status. The English and South African Afrikaans and isiXhosa language versions of the EQ5D, were previously validated and accepted by the European Quality of Life Group (Jelsma & Ferguson 2004; Jelsma et al. 2004). This self-reporting measure was also found to be valid and reliable in the stroke population (Hunger et al. 2012).

The 10-item BI was utilised as a self-report measure to assess independence in ADL in the current study. The BI is considered the gold standard in measuring functional independence in stroke patients and has excellent validity and reliability (Salter et al. 2013). Each item of the BI was scored between 0 and 10, with a maximum ideal total score of 100. A higher score indicated a higher degree of independence (Salter et al. 2013). The mRS is a frequently used scale to assess stroke severity or level of disability (Kasner 2006) and was used for this purpose in the study. The mRS has excellent validity in the assessment of stroke patients but lacks reliability in this population (Kasner 2006). Patients are given a score of 0–6; 0 being no symptoms, 1 indicating no disability despite symptoms, 2 slight disability, 3 moderate disability, 4 moderately severe disability, 5 indicating severe disability, and 6 death (Bonita & Beaglehole 1988; Rankin 1957).

Once all data were coded and captured in Microsoft Excel, statistical analysis was done. Continuous data including BI and mRS were summarised using median, interquartile range (IQR) and empirical 95% confidence intervals (CI). Statistical analysis was performed using Statistical Package for the Social Sciences (SPSS) version 25. Association between categorical variables was assessed using the Wilcoxon signed ranks test. Differences in distribution of continuous variables over different levels of categorical variables were evaluated using the Kruskal-Wallis test, and where differences were detected, the Dunn’s test was used for pairwise comparisons. Statistical significance was assessed at the 5% level. Correlations were interpreted as follows: 0.20 – 0.39 = weak; 0.40 – 0.59 = moderate; 0.60 – 0.79 = strong; and 0.80 – 1.00 = very strong (Mukaka 2012).

## Results

### Demographics

As summarised in Table 1, the median age of participants was 48 years, and they had a mean length of stay of 53 days. Majority of participants had an infarction-type stroke (89.90%) with a nearly equal number of participants presenting with left- and right-sided hemiplegia. Hypertension and diabetes were the most common stroke risk factors (73.47% and 28.57%, respectively), as well as a low cluster of differentiation 4 (CD4) count in participants with HIV+ diagnosis (*n* = 9; median CD4 count: 130).

**TABLE 1:** Demographic and stroke related characteristics.

Variables	Values				
	*n*	%	Median	IQR	Range
**Sample size**					
Admission	49	-	-	-	-
Discharge	41	-	-	-	-
**Age**	-	-	48	39–60	-
Women	25	51.02	-	-	-
**Type of stroke**					-
Infarction	44	89.80	-	-	-
Right CVA (Left hemiplegia)	25	51.02	-	-	-
**Risk factors for stroke**					-
No known risk factor	2	4.08	-	-	-
Hypertension	36	73.47	-	-	-
Diabetes	14	28.57	-	-	-
Cholesterol	6	12.24	-	-	-
Smoking	16	32.65	-	-	-
Substance abuse	3	6.12	-	-	-
Opportunistic infections	3	6.12	-	-	-
HIV-1 infection	9	18.37	-	-	-
CD4 count, mdn (range)	-	-	130	-	54–883
**Time (days) between stroke incident and admission, median (IQR)**	-	-	20	14–28	-
**Length of stay (days), median (IQR)**	-	-	53	46–60	-

*Source:* Hartley, T., Burger, M., Esterhuizen, T.M. & Inglis-Jassiem, G., 2020, ‘Functional outcome of stroke inpatients according to human immunodeficiency virus status: A feasibility study’, *African Journal of Disability* 9(0), a618. https://doi.org/10.4102/ajod.v9i0.618

Mdn, median; IQR, interquartile range; *n*, number; CVA, cerebrovascular accident.

### Functional status and stroke severity

The biggest improvement noted on discharge in the mRS measure was the decrease in the number of participants scoring 4; that is, presenting with moderately severe disability, being unable to walk without assistance nor being able to attend to own bodily needs without assistance. On admission, a total of 63.27% (*n* = 31) scored 4 and because of improvement, this number significantly reduced to 26.83% (*n* = 11) on discharge with the median score for the sample being 2 (*p* < 0.001). A score of 2 indicated slight disability with participants being unable to carry out all previous activities but able to look after own affairs without assistance. On discharge, 31.71% (*n* = 13) of participants scored 1, which is indicative of no significant disability despite symptoms, which indicates that participants were able to carry out all usual duties and activities (see Table 2). Although these results proved statistically significant, it is important to note that the mRS has neither established minimally clinically important difference (MCID) nor minimal detectable change (MDC) (De Haan et al. 1995).

**TABLE 2:** Stroke severity measured with Modified Rankin Scale.

Level	Admission total *n* = 49		Discharge total *n* = 41		Median difference	IQR	Wilcoxon Signed Rank test, test-statistic	*p*
	*n*	%	*n*	%				
1	0	0.00	13	31.71	−1	−2, 0	4.796	*p* < 0.001*
2	7	14.29	9	21.95	-	-	-	-
3	9	18.37	7	17.07	-	-	-	-
4	31	63.27	11	26.83	-	-	-	-
5	2	4.08	1	2.44	-	-	-	-
Median	4		2		-	-	-	-
IQR	3–4		1–4		-	-	-	-

*n*, number; IQR, interquartile range.

* , indicates statistical significance.

On admission, the median BI score of 55 indicated severe dependence in ADL or maximal assistance required with self-care and mobility. The median BI score improved by 35 points on discharge (median 90; *p* < 0.001) indicating that some assistance was still required with mobility for a few participants (see Table 3). The BI has a MDC in score of 4.02 points (Hsieh et al. 2007), hence the current sample of participants demonstrated both a statistically and clinically significant functional improvement during inpatient rehabilitation. The changes in score on discharge showed a very strong negative significant correlation between the mRS and the BI (rs = -0.874 *p* < 0.001) (see Table 4).

**TABLE 3:** Functional status measured with Barthel Index ß.

Variables	Admission total *n* = 49	Discharge total *n* = 41	Median difference (IQR)	Wilcoxon Signed Rank test	*p*
Median	55	90	35	5.366	*p* < 0.001*
IQR	40–75	40–75	10–45	-	-

*n*, number; IQR, interquartile range.

* , indicates statistical significance; ß Barthel Index total score is 100, a higher score indicates higher degree of independence in ADL.

**TABLE 4:** Correlation between Euro-QoL Five Dimensions instrument Visual Analog Scale score, modified Rankin Scale and Barthel Index scores.

Outcome measure correlations	Admission scores	Discharge scores
**EQ5D vs mRS**		
Correlation coefficient	−0.073	−0.362*
*p*	0.617	0.02
**EQ5D vs BI**		
Correlation coefficient	0.111	0.329*
*p*	0.449	0.036
**mRS vs BI**		
Correlation coefficient	−0.672**	−0.874****
*p*	< 0.001	< 0.001

EQ5D, Euro-QoL Five Dimensions instrument; BI, Barthel Index.

* , indicates weak correlation;

** , indicates strong correlation;

*** , indicates strong correlation;

**** , indicates very strong correlation.

### Health related quality of life

The median score for the best imaginable health state on admission (measured on VAS), increased by 30 on discharge indicating a significant improvement post rehabilitation (refer to Table 5). In terms of the EQ5D item descriptors, level 1 is better than a level 2, hence a negative median difference in score would indicate an improvement. All domains except anxiety/depression showed a significant improvement in their median difference score between admission and discharge (refer to Table 5). In comparison to admission, participants improved significantly in the functional domains of mobility, self-care and usual activity with majority of participants reporting no problems in each of these domains upon discharge (*p* < 0.001). Participants experiencing no symptoms of pain/discomfort improved from 57.14% to 68.29% on discharge. Even though the median score for the pain/discomfort domain was 0, showing no improvement, seven participants improved from a 2 to a 1 score, whereas one participant changed from a score of 1–2, hence an overall statistically significant improvement was noted (*p* = 0.034). With regards to the anxiety/depression domain, 65.31% of participants reported no symptoms of anxiety/depression on admission compared to 78.05% reporting no symptoms on discharge. Although this domain of anxiety/depression had a median change in score of 0, a total of nine participants improved from a score of 2 to 1, whereas four regressed from a score of 1 to a 2. Hence, no statistically significant findings were noted (*p* = 0.378). On discharge the changes in score showed a weak negative significant correlation between the EQ5D VAS score and the mRS (rs = -0.362; *p* = 0.02). A weak positive significant correlation was seen between the EQ5D VAS score and the BI (rs = 0.329; *p* = 0.036) (see Table 4).

**TABLE 5:** Health-related QoL measured with Euro-QoL Five Dimensions instrument.

Domain	Levelø	Admission total *n* = 49		Discharge total *n* = 41		Median difference	IQR	Wilcoxon Signed Rank test, test-statistic	*p*
		*n*	%	*n*	%				
**EQ5D best imaginable health state scale measured by VAS**									
EQ5D VAS median				-	-	30	12.5, 40.0	5.289	*p* < 0.001*
Median	50			80		-	-	-	-
IQR	40.70			70.90		-	-	-	-
**EQ5D domains**									
Mobility	1†	3	6.12	21	51.22	−1	−1, 0	4.491	*p* < 0.001*
	2‡	42	85.71	20	48.80	-	-	-	-
	3¶	4	8.16	0	0.00	-	-	-	-
Median	2			1		-	-	-	-
IQR	2-2			1–2		-	-	-	-
Self-care	1†	6	12.24	32	78.05	−1	−1.0, 0	5.112	*p* < 0.001*
	2‡	42	85.71	9	21.95	-	-	-	-
	3¶	1	2.04	0	0.00	-	-	-	-
Median	2			1		-	-	-	-
IQR	2–2			1–1		-	-	-	-
Usual activities	1†	3	6.12	24	58.54	−1	−1.5, -0.5	5.072	*p* < 0.001*
	2‡	24	48.98	16	39.02	-	-	-	-
	3¶	22	44.90	1	2.44	-	-	-	-
Median	2			1		-	-	-	-
IQR	2–3			1–2		-	-	-	-
Pain/discomfort	1†	28	57.14	28	68.29	0	0, 0	2.121	*p* = 0.034*
	2‡	20	40.82	12	29.27	-	-	-	-
	3¶	1	2,04	1	2.44	-	-	-	-
Median	1			1		-	-	-	-
IQR	1–2			1–2		-	-	-	-
Anxiety/depression	1†	32	65.31	32	78.05	0	0,0	0.881	*p* = 0.378
	2‡	14	28.57	8	19.51	-	-	-	-
	3¶	3	6.12	1	2.44	-	-	-	-
Median	1			1		-	-	-	-
IQR	1–2			1–1		-	-	-	-

*n*, number; VAS, Visual Analog Scale; IQR, interquartile range; EQ5D, Euro-QoL Five Dimensions instrument.

* , indicates statistical significance;

ø , EQ5D level ranges from 1 to 3;

† , being the more favourable category indicating no problems/no symptomsl;

‡ , some problems/ moderate symptoms;

¶ , unable to perform task/severe symptoms in the respective domain.

## Discussion

The significant improvements in BI and mRS scores upon discharge showed great improvement in functional independence and severity in disability. The negative correlation between the BI and the mRS in the current study was expected as participants’ stroke severity score (mRS) would decrease as they became more independent in ADL, while BI would oppositely increase as function improved. Thus, the very strong correlation (rs = -0.874) may indicate participants’ stroke severity decreased to a similar degree to which their independence in ADL improved (Langhammer et al. 2017).

Similarly, a significant improvement was seen in all the functional domains (i.e. mobility, self-care, usual activities) of the EQ5D with most participants scoring in the higher percentiles for these domains. However, pain/discomfort showed a significant improvement, but not to the same degree as functional domains. No significant change was seen with regards to the anxiety/depression domain which may indicate this domain did not improve to the same degree. Functional ability greatly influences HRQoL (Abubakar & Isezuo 2012; Delcourt et al. 2011; Howitt et al. 2011; Raju, Sarma & Pandian 2010). Stroke survivors with more severe neurological impairments and therefore more residual physical disability post stroke may require long-term care and assistance with ADL, thus significantly reducing their HRQoL (Bettger et al. 2014). Previous literature has shown that therapies such as physiotherapy and occupational therapy improve physical functioning, and hence leads to improvement in overall HRQoL (Christian & Fink 2020; Langhammer et al. 2017).

However, the weak correlations between the EQ5D and functional measures BI and mRS shows that even though stroke severity decreased and functional independence increased, the improvement in EQ5D VAS score/perception of best imaginable health state did not improve to the same degree. Some studies also report poor HRQoL post stroke even with minimal to no disability (Lai et al. 2002), while others identified functional status as an independent factor affecting HRQoL (Abubakar & Isezuo 2012; Katona et al. 2015). A poorer perceived HRQoL even with significant improvement in functional status may be affected by age (Abubakar & Isezuo 2012) where having a stroke at a younger age may have devastating effects (Hartley et al. 2020).

Because of the general occurrence of musculoskeletal impairments and neuropathic pain post stroke, pain and discomfort is often a common occurrence in stroke survivors and can affect functional ability (Benlidayi & Basaran 2013; Kong et al. 2004). Thus, this domain may not have improved to the same degree as functional domains. In a 2.5-year prospective longitudinal study by Katona et al. (2015), the pain/discomfort domain in the EQ5D deteriorated twice as much as compared to other EQ5D domain improvements seen over time. Previous studies report that up to 70% of stroke patients suffer from chronic pain (Harrison & Field 2015; Naess, Lunde & Brogger 2012). This is concerning as chronic pain may not only impair functional ability and increase the risk of depression, but further reduce HRQoL and add to the anxiety patients may experience post stroke (Benlidayi & Basaran 2013; Harrison & Field 2015; Katona et al. 2015; Kong et al. 2004). Moreover, even though there is sufficient evidence for effective treatment for pain post stroke, previous literature found that patients are often not diagnosed, not given the appropriate treatment, or not treated at all (Langhorne et al. 2000; Widar et al. 2002).

Anxiety/depression was the only domain on the EQ5D to not demonstrate a significant improvement. Anxiety and depression should not be overlooked as these conditions have been closely correlated with poorer physical and psychological HRQoL in other stroke survivors (Howitt et al. 2011). A systematic review reports that 1 in 3 stroke survivors in Sub-Saharan Africa suffers from depression (Ojagbemi et al. 2017b). In addition, 1 in 5 stroke survivors in Sub-Saharan Africa suffer from clinical anxiety and more than 70% of those suffering from anxiety also have depression (Ojagbemi et al. 2017b). Abubakar and Isezuo (2012) conducted a cross-sectional correlation descriptive study on 62 patients 3-months post stroke, measuring factors influencing HRQoL which included depression measured by the Zung depression self-rating scale. The authors found depression to be an independent determinant of HRQoL in stroke survivors (Abubakar & Isezuo 2012). Other studies, reporting the negative effects of anxiety and depression on HRQoL, demonstrated that anxiety had a greater impact on psychosocial issues as compared to physical health (Donnellan et al. 2010; Morris et al. 2013; Raju et al. 2010; Tang et al. 2013). Anxiety and depression post stroke further reduces return to usual activities, which may result in loss of productivity. This may cause added personal and social losses (Chen et al. 2019; Harris 2014). The authors report that anxiety symptoms may take a while to appear and usually occur between 1 month and 1 year post incident. Lynch et al. (2020) reported on the long-term outcomes of stroke patients discharged into an inpatient facility compared to patients being discharged home from acute care. The study found that patients discharged home had a poorer HRQoL which may be because of less support and increased physical demands (Chen et al. 2019; Harris 2014). However, Kainz et al. (2021) conducted a 12 month follow up of stroke patients and found that patients had a better HRQoL at 12 months as compared to their 3-month follow up. Although, these participants living with stroke had a poorer reported HRQoL as compared to the healthy age-matched population at 12 months. Hence, a longer-term approach is needed in order to diagnose, monitor and treat these mental health-related symptoms to improve overall HRQoL (Katona et al. 2015).

Being that the current median age for our sample was 48 years, the consequences of stroke may be more devastating for these younger participants who are at risk of not returning to full function or resume participation and productive activity. As at this age, individuals would most likely be involved in work and/or be responsible for dependents. Young stroke occurring in HIV positive (+) people has become a trend in current literature and was a notable concern in the current study sample (Hartley et al. 2020; Heikinheimo et al. 2012; Taylor & Ntusi 2019).

The younger cohort of stroke survivors in the current study may reflect other socioeconomic and physical challenges as their day-to-day activities may have required more responsibilities. Palmcrantz et al. (2014) conducted a cross-sectional study in Sweden comparing HRQoL of young strokes to the general population using the EQ5D. When comparing age and geographically matched groups, the young stroke group rated themselves significantly lower in most of the domains on the EQ5D except the pain/discomfort domain. Even though young strokes are expected to make a better recovery than their older counter parts, family responsibilities and return to work (RTW) may be greatly affected. However, other studies such as the Nigerian study conducted by Abubakar and Isezuo (2012) found that age had no negative influence on HRQoL.

When comparing the current study sample VAS scores (80) to scores of older persons living with stroke (60–75 years), the current study sample had a better outcome as would be expected with a younger stroke population (Lynch et al. 2020). However, a systematic review conducted on studies with African stroke survivors found that this population had a poorer HRQoL as compared to their healthy age-matched counter parts (Bello et al. 2021). Similar results were found in a South African study which utilised the EQ5D (Jelsma & Ferguson 2004). The general public had a higher percentage of participants who reported no problems for all functional domains but less so for pain/discomfort and anxiety/depression. The general public also had a higher median VAS score (85.1) as compared to the current study population on discharge (80) (Jelsma & Ferguson 2004). With regards to the pain/discomfort and anxiety/depression domains as well as VAS, the current study’s sample may have scored similar or higher than the general public as a result of not permanently residing at home, not being age matched and having more support within the inpatient rehabilitation setting. Rhoda (2014), who conducted a similar study in the Western Cape but with community dwelling stroke survivors, found that people with stroke did poorer in all domains of the EQ5D compared to the general public. International studies had similar findings to Rhoda (2014), hence these findings highlight the negative impact stroke may have on function, pain, mental health and overall HRQoL (Naess et al. 2006; Unibaso-Markaid et al. 2019).

Quality of life extends to productivity or return to previous function such as work. Westerlind et al. (2017) conducted a 6-year follow up study on RTW of stroke participants under 63 years in Sweden. These authors linked degree of disability on discharge as a potential predictor for RTW post stroke. The authors categorised a score of 0–2 on the mRS as functional independency and 3–6 as dependency. Those participants who scored 0–2 on the mRS were likely to RTW and those who scored between 3 and 6 in addition to being on sick leave prior to stroke, were not likely to RTW (Westerlind et al. 2017). At the 3-year mark, 48.3% of participants RTW and at 6 years 74.7% of participants RTW. With regards to EQ5D domains, no significant differences were seen between those who RTW and those who did not. However, a significant difference was seen with regards to the EQ5D VAS scores (*p* = 0.012) with those who RTW rating a higher overall HRQoL (Westerlind et al. 2017). This study demonstrated a high RTW rate, most likely because of the Swedish government providing subsidies to companies who employ disabled individuals, in addition to being a high-income country with tax funded rehabilitation, care and sick leave. Even though the current study sample had a median of 2 on the mRS, South Africa is a middle-income country. Therefore, in the South African context, the support given by the government in addition to education levels, socio-economic factors, limited resources and ill equipped health system may negatively influence RTW and overall HRQoL for people with stroke (De la Cornillere 2007; Kahonde, Mlenzana & Rhoda 2010; Kumurenzi et al. 2015; National Health Insurance 2011; Ntsiea 2019; Sulla & Zikhali 2018). Palmcrantz et al. (2014) found that in addition to RTW in young strokes, leisure activities, which is used to cope with stroke deficits, were associated with physical health and should become an integral part of rehabilitation programmes (Carlsson, Moller & Blomstrand 2009; Vestling et al. 2003). The findings of this study and previous literature infer that rehabilitation and future studies should focus on psychosocial aspects of health and mitigating social barriers to achieve full community reintegration (Alguren et al. 2012).

In terms of the South African context, there has been a rapid change in the stroke demographic which Taylor and Ntusi (2019) referred to as an epidemiological transition. Given that global trends indicate an increase in a younger stroke population, rehabilitation services should ideally cater for more than safety and independence in ADL (Boot et al. 2020; Guise et al. 2010; Ntsiea 2019). Previous studies conducted on stroke rehabilitation in South Africa report that stroke survivors are discharged prematurely because of limited rehabilitation services and have a poor recovery rate post discharge from inpatient rehabilitation (Ntsiea 2019; Scheffler & Mash 2019). Rehabilitation was often focused on functional independence to participate within community with little emphasis being placed on barriers limiting full social integration and psychosocial aspects (Duff 2012; Guise et al. 2010; Ntsiea et al. 2013; Scheffler & Mash 2019). In addition, stroke patients were further limited in terms of education, work and transport upon discharge (Ntsiea 2019). Furthermore, accessibility to community rehabilitation services or home-based care are limited on account of poor resources, transportation difficulties, poor referral systems and lack of information about rehabilitation services (De la Cornillere 2007; Kahonde et al. 2010; Kumurenzi et al. 2015; Ntsiea 2019; Scheffler & Mash 2019).

Thus, regardless of the functional recovery the current study sample has gained within inpatient rehabilitation, if this is not followed through with adequate community rehabilitation aimed at improving the patients perceived HRQoL, their anxiety/depression as well as pain/discomfort may further deteriorate. This is a great challenge as majority of South Africans use the public healthcare system which is riddled with inequality in access and distribution of healthcare resources, including support and mental healthcare services (National Health Insurance 2011).

## Limitations

With regards to the limitations in the current study, the selection bias (by excluding participants with severe communication disorders), the small sample size, recruitment from one site, and short follow up period, hindered generalisability of results. It is unclear whether HRQoL may have regressed on return home as participants no longer had the support of the specialist inpatient facility. In addition, even though the EQ5D includes the domain of usual activities, referring to work and study, specific statistics on return to productive activity were not included in the current study nor were participants followed up after discharge.

## Recommendations

We recommend that future studies include 6–12 month follow up periods to attain information on residential and community reintegration as well as return to productive activity and how this may impact HRQoL. In addition, a probability sampling method for future studies could be used to improve generalisation of results. Clinical outcomes within the rehabilitation setting need to accommodate for the new stroke demographic and potentially longer length of stay to optimise recovery to include community reintegration which includes participating in civic life, work and education (WHO 2011).

In addition, we recommend that clinicians strategically select outcome measures to monitor the person with stroke’s perception of HRQoL (and potentially the long-term sequelae of pain/discomfort and anxiety and depression). We advocate that these person-centred outcomes should be routinely incorporated in stroke rehabilitation- and discharge planning along the continuum of care from acute care to later stages of community reintegration. These intervention and monitoring strategies should form integral parts of goal setting during post-stroke rehabilitation to foreground patient and family support needs and where indicated, earlier referral to psychologists and/or community-based stroke support groups.

## Conclusion

Although an improvement was noted in HRQoL of people with stroke receiving inpatient rehabilitation, their EQ5D VAS scores tended not to improve as strongly, despite significant improvements in function and stroke severity. These findings demonstrate the need for psychological support and pain management interventions for the post-stroke adjustment. As these physical and psychosocial needs may only become more pertinent once people with stroke are home and discharged from acute or inpatient rehabilitation settings, support services and long-term self-management interventions could be better placed, and therefore more accessible, at a community level. Implementation of these community-based support services may be the key to better quality of life and realising productive activity for younger stroke survivors in South Africa.

## References

[CIT0001] Abubakar, S.A. & Isezuo, S.A., 2012, ‘Health related quality of life of stroke survivors: Experience of a stroke unit’, *International Journal of Biomedical Sciences* 8(3), 183–187. PMC3615283 23675271

[CIT0002] Alguren, B., Fridlund, B., Cieza, A., Sunnerhagen, K.S. & Christensson, L., 2012, ‘Factors associated with health- related quality of life after stroke: A 1-year prospective cohort study’, *Neurorehabilitation and Neural Repair* 26(3), 266–274. 21825005 10.1177/1545968311414204

[CIT0003] Badaru, U.M., Omoyemi, O. & Ade, A, 2015, ‘Health related quality of life of stroke survivors in Africa: A critical review of literature’, *Archives of Physiotherapy and Global Research* 19(3), 7–16. 10.15442/apgr.19.2.13

[CIT0004] Bello, U.M., Chutiyami, M., Salihu, D., Abdu, S.I., Tafida, B.A., Jabbo, A.A. et al., 2020, ‘Quality of life of stroke survivors in Africa: A systematic review and meta-analysis’, *Quality of Life Research* 30(1), 1–19. 10.1007/s11136-020-02591-6 32712933

[CIT0005] Benlidayi, C. & Basaran, I., 2013, ‘Hemiplegic shoulder pain: A common clinical consequence of stroke’, *Practical Neurology* 14(2), 88–91. 10.1136/practneurol-2013-000606 23940374

[CIT0006] Bertram, M.Y., Katzenellenbogen, J., Vos, T., Bradshaw, D. & Hofman, K.J., 2013, ‘The disability adjusted life years due to stroke in South Africa in 2008’, *International Journal of Stroke* 8(Suppl A100), 76–80. 10.1111/j.1747-4949.2012.00955.x 23295022

[CIT0007] Bettger, J.P., Zhao, X., Bushnell, C., Zimmer, L., Pan, W., Williams, L.S. et al., 2014, ‘The association between socioeconomic status and disability after stroke: Findings from the Adherence eValuation After Ischemic stroke Longitudinal (AVAIL) registry’, *BMC Public Health* 14, 281. 10.1186/1471-2458-14-281 24666657 PMC3987648

[CIT0008] Bonita, R. & Beaglehole, R., 1988, ‘Modified Randkin Scale: Recovery of motor function after stroke’, *Stroke* 19(12), 1497–1500. 10.1161/01.STR.19.12.1497 3201508

[CIT0009] Boot, E., Ekker, M.S., Putaala, J., Kittner, S., De Leeuw, F.E. & Tuladhar, A.M., 2020, ‘Ischaemic stroke in young adults: a global perspective’. *Journal of Neurology, Neurosurgery & Psychiatry* 91(4), 411–417. 10.1136/jnnp-2019-322424 32015089

[CIT0010] Bryer, A., Connor, M., Haug, P., Cheyip, B., Staub, H., Tipping, B. et al., 2010, ‘South African guideline for management of ischaemic stroke and transient ischaemic attack 2010: A guideline from the South African Stroke Society (SASS) and the SASS Writing Committee’, *South African Medical Journal* 100(11), 747–778. 10.7196/SAMJ.4422 21081029

[CIT0011] Carlsson, G.E., Moller, A. & Blomstrand, C., 2009, ‘Managing an everyday life of uncertainty – A qualitative study of coping in persons with mild stroke’, *Disability & Rehabilitation* 31(10), 773–782. 10.1080/09638280802638857 19350431

[CIT0012] Chen, Q., Cao, C., Gong, L. & Zhang, Y., 2019, ‘Health related quality of life in stroke patients and risk factors associated with patients for return to work’, *Medicine* 98(16), e15130. 10.1097/MD.0000000000015130 31008934 PMC6494282

[CIT0013] Christian, G. & Fink, G.R., 2020, ‘Recovery from stroke: Current concepts and future perspectives’, *Neurological Research and Practice* 2, 17. 10.1186/s42466-020-00060-6 33324923 PMC7650109

[CIT0014] De Haan, R.J., Limburg, M., Bossuyt, P., Van der Meulen, J. & Aaronson, N., 1995, ‘The clinical meaning of Rankin “handicap” grades after stroke’, *Stroke* 26(11), 2027–2030. 10.1161/01.STR.26.11.2027 7482643

[CIT0015] De la Cornillere, W.L., 2007, ‘Participants’ experiences of the Bishop Lavis Rehabilitation Centre stroke group, Master’s thesis’, Department of Interdisciplinary Health Sciences, Stellenbosch University.

[CIT0016] Delcourt, C., Hacket, M., Wu, Y., Huang, Y., Wang, J., Heeley, E. et al., 2011, ‘Determinants of quality of life in China: The China QUEST (Quality Evaluation of Stroke and Treatment) Study’, *Stroke* 42, 433–438. 10.1161/STROKEAHA.110.596627 21183752

[CIT0017] Donnellan, C., Hickey, A. & Hevey, D. & O’Neill, D., 2010, ‘Effect of mood symptoms on recovery one year after stroke’, *International Journal of Geriatric Psychiatry* 25(12), 1288–1295. 10.1002/gps.2482 21086539

[CIT0018] Dorman, P. J., Waddell, F., Slattery, J., Dennis, M. & Sandercock, P., 1997, ‘Is the EuroQol a valid measure of health-related quality of life after stroke?’, *Stroke* 28(10), 1876–1882. 10.1161/01.STR.28.10.1876 9341688

[CIT0019] Duff, N., 2012, *Perceived factors that influence return to work after stroke*, Unpublished research report, Faculty of Health Sciences, University of the Witwatersrand, Johannesburg.

[CIT0020] Guise, J., McKinlay, A., Widdicombe, A., 2010, ‘The impact of early stroke on identity: A discourse analytic study’, *Health* 14, 75–90. 10.1177/1363459309347483 20051431

[CIT0021] Harris, C., 2014, ‘Return to work after stroke: A nursing state of the science’, *Stroke* 45, e174–e176. 10.1161/STROKEAHA.114.006205 25013019

[CIT0022] Harrison, R.A. & Field, T.S., 2015, ‘Post stroke pain: Identification, assessment, and therapy’, *Cerebrovascular Diseases* 39, 190–201. 10.1159/000375397 25766121

[CIT0023] Hartigan, I., O’Connell, E., McCarthy, G. & O’Mahony, D., 2011, ‘First time stroke survivors’ perceptions of their health status and their goals for recovery’, *International Journal of Nursing and Midwifery* 3(2), 22–29.

[CIT0024] Hartley, T., Burger, M., Esterhuizen, T.M. & Inglis-Jassiem, G., 2020, ‘Functional outcome of stroke inpatients according to human immunodeficiency virus status: A feasibility study’, *African Journal of Disability* 9(0), a618. 10.4102/ajod.v9i0.618 PMC7136685 32284924

[CIT0025] Heikinheimo, T., Chimbayo, D., Kumwenda, J., Kampondeni, S. & Allain, T.J., 2012, ‘Stroke outcomes in Malawi, a country with high prevalence of HIV: A prospective follow-up’, *PLoS One* 7(3), e33765. 10.1371/journal.pone.0033765 22479439 PMC3315584

[CIT0026] Howitt, S.C., Jones, M.P., Jusabani, A., Gray, W.K., Aris, E., Mugusi, F. et al., 2011, ‘A cross-sectional study of the quality of life in incident stroke survivors in rural northern Tanzania’, *Journal of Neurology* 258(8), 1422–1430. 10.1007/s00415-011-5948-6 21336782

[CIT0027] Hsieh, Y., Wang, C., Wu, S., Chen, P., Sheu, C. & Hsieh, C., 2007, ‘Establishing the minimal clinically important difference of the Barthel Index in stroke patients’, *Neurorehabilitation and Neural Repair* 21(3), 233–238. 10.1177/1545968306294729 17351082

[CIT0028] Hunger, M., Sabariego, C., Stollenwerk, B., Cieza, A. & Leidl, R., 2012, ‘Validity, reliability and responsiveness of the EQ5D in German stroke patients undergoing rehabilitation’, *Quality of Life Research* 21(7), 1205–1216. 10.1007/s11136-011-0024-3 21971874

[CIT0029] Isaac, V., Stewart, R. & Krishnamoorthy, E.S., 2010, ‘Caregiver burden and quality of life of older persons with stroke’, *Journal of Applied Gerontology* 30(5), 643–654. 10.1177/0733464810369340

[CIT0030] Jalali, R. & Dutta, D., 2012, ‘Factors influencing quality of life in adult patients with primary brain tumors’, *Neuro Oncology* 14(4), iv8–iv16. 10.1093/neuonc/nos205 23095834 PMC3480247

[CIT0031] Jelsma, J. & Ferguson, G., 2004, ‘The determinants of self-reported health-related quality of life in a culturally and socially diverse South African community’, *Bulletin of the World Health Organization* 82, 206–212. 15112009 PMC2585936

[CIT0032] Jelsma, J., Mkoka, S., Amosun, L. & Nieuwveldt, J., 2004, ‘The reliability and validity of the Xhosa version of the EQ-5D’, *Disability and Rehabilitation* 26(2), 103–108. 10.1080/09638280310001629705 14668147

[CIT0033] Kahonde, C.K., Mlenzana, N. & Rhoda, A., 2010, ‘Persons with physical disabilities experiences of rehabilitation services’, *South African Journal of Physiotherapy* 30(4), 877–879. 10.4102/sajp.v66i3.67

[CIT0034] Kainz, A., Meisinger, C., Linseisen, J., Kirchberger, I., Zickler, P., Naumann, M. et al., 2021, ‘Changes of health-related quality of life within the 1st year after stroke–results from a prospective stroke cohort study’, *Frontiers in Neurology* 12, 715313. 10.3389/fneur.2021.715313 34671308 PMC8520951

[CIT0035] Kasner, S.E., 2006, ‘Clinical interpretation and use of stroke scales’, *The Lancet Neurology* 5(7), 603–612. 10.1016/S1474-4422(06)70495-1 16781990

[CIT0036] Katona, M., Schmidt, R., Schupp, W. & Graessel, E., 2015, ‘Predictors of health-related quality of life in stroke patients after neurological inpatient rehabilitation: A prospective study’, *Health and Quality of Life Outcomes* 3, 58. 10.1186/s12955-015-0258-9 PMC4448207 25971254

[CIT0037] Kengne, A.P. & Anderson, C.S., 2006, ‘The neglected burden of stroke in sub-Saharan Africa’, *International Journal of Stroke: Official Journal of the International Stroke Society* 1(4), 180–190. 10.1111/j.1747-4949.2006.00064.x 18706015

[CIT0038] Kong, K., Woon, V. & Yang, S.Y., 2004, ‘Prevalence of chronic pain and its impact on health-related quality of life in stroke survivors’, *Archives of Physical Medicine and Rehabilitation* 85(1), 35–40. 10.1016/S0003-9993(03)00369-1 14970965

[CIT0039] Kumurenzi, A., Goliath, C., Mji, G., Mlenzana, N., Joseph, C., Stathum, S. et al., 2015, ‘Experiences of patients and service providers with out- patient rehabilitation services in a rehabilitation centre in the Western Cape Province’, *African Journal of Disability* 4(1), Art. #164, 7 pages. 10.4102/ajod.v4i1.164 PMC5433475 28730027

[CIT0040] Lai, M.S., Studenski, S., Duncan, P.W. & Perera, S., 2002, ‘Persisting consequences of stroke measured by the stroke impact scale’, *Stroke* 33, 1840–1844. 10.1161/01.STR.0000019289.15440.F2 12105363

[CIT0041] Langhammer, B., Sunnerhagen, K.S., Lundgren-Nilsson, Å., Sällström, S., Becker, F. & Stanghelle, J.K., 2017, ‘Factors enhancing activities of daily living after stroke in specialized rehabilitation: An observational multicenter study within the Sunnaas International Network’, *European Journal of Physical and Rehabilitation Medicine* 53(5), 725–734. 10.23736/S1973-9087.17.04489-6 28417611

[CIT0042] Langhorne, P., Bernhardt, J. & Kwakkel, G., 2011, ‘Stroke rehabilitation’, *Lancet* 377(9778), 1693–1702. 10.1016/s0140-6736(11)60325-5 21571152

[CIT0043] Langhorne, P., Stott, D.J., Robertson, L., MacDonald, J., Jones, L., McAlpine, C. et al., 2000, ‘Medical complications after stroke: A multicenter study’, *Stroke* 31, 1223–1229. 10.1161/01.STR.31.6.1223 10835436

[CIT0044] Lozano, R., Naghavi, M., Foreman, K., Lim, S., Shibuya, K., Aboyans, V. et al., 2012, ‘Global and regional mortality from 235 causes of death for 20 age groups in 1990 and 2010: A systematic analysis for the Global Burden of Disease Study 2010’, *Lancet* 380(9859), 2095–2128. 10.1016/S0140-6736(12)61728-0 23245604 PMC10790329

[CIT0045] Lynch, E., Labberton, A.S., Kim, J. & Kilkenny, M., 2020, ‘Out of sight, out of mind: Long-term outcomes for people discharged home, to inpatient rehabilitation and to residential aged care after stroke’, *Disability and Rehabilitation* 14, 1–7. 10.1080/09638288.2020.1852616 33307842

[CIT0046] Maredza, M., Bertram, M.Y., Gómez-Olivé, X.F. & Tollman, S.M., 2016, ‘Burden of stroke attributable to selected lifestyle risk factors in rural South Africa’, *BMC Public Health* 1(16), 143. 10.1186/s12889-016-2805-7v PMC4751665 26869067

[CIT0047] Maredza, M., Bertram, M.Y. & Tollman, S.M., 2015, ‘Disease burden of stroke in rural South Africa: An estimate of incidence, mortality and disability adjusted life years’, *BMC Neurology* 15(1), 54. 10.1186/s12883-015-0311-7 25880843 PMC4396076

[CIT0048] Markus, H., 2012, ‘Stroke: Causes and clinical features’, *Medicine* 40(9), 484–489. 10.1016/j.mpmed.2012.06.005

[CIT0049] Morris, J.H., Van Wijck, F., Joice, S. & Donaghy, M., 2013, ‘Predicting health related quality of life 6 months after stroke: The role of anxiety and upper limb dysfunction’, *Disability and Rehabilitation* 35(4), 291–299. 10.3109/09638288.2012.691942 22691224

[CIT0050] Mukaka, M.M., 2012, ‘Statistics corner: A guide to appropriate use of correlation coefficient in medical research’, *Malawi Medical Journal: The Journal of Medical Association of Malawi* 24(3), 69–71, viewed 22 October 2018, from http://www.ncbi.nlm.nih.gov/pubmed/23638278. 23638278 PMC3576830

[CIT0051] Naess, H., Lunde, L. & Brogger, J., 2012, ‘The effects of fatigue, pain, and depression on quality of life in ischemic stroke patients: The Bergen stroke study’, *Vascular Health and Risk Management* 8, 407–413. 22910531 10.2147/VHRM.S32780 PMC3402053

[CIT0052] Naess, H., Waje-Andreassen, U., Thomassen, L. & Nyland, H., 2006, ‘Health-related quality of life among young adults with ischemic stroke on long-term follow-up’, *Stroke* 37, 1232–1236. 10.1161/01.STR.0000217652.42273.02 16601213

[CIT0053] Naghavi, M., Abajobir, A.A., Abbafati, C., Abbas, K.M., Abd-Allah, F., Abera, S.F. et al., 2017, ‘Global, regional, and national age-sex specific mortality for 264 causes of death, 1980–2016: A systematic analysis for the Global Burden of Disease Study 2016’, *Lancet* 390, 1151–1210. 28919116 10.1016/S0140-6736(17)32152-9 PMC5605883

[CIT0054] National Health Insurance, 2011, *Government Gazette number 3423*, 12 August 2011, p. 8.

[CIT0055] Ntsiea, M.V., 2019, ‘Current stroke rehabilitation services and physiotherapy research in South Africa’, *South African Journal of Physiotherapy* 75(1), a475. 10.4102/sajp.v75i1.475 PMC6676941 31392288

[CIT0056] Ntsiea, M.V., Van Aswegen, H. & Olorunju, S., 2013, ‘Factors which are predictive of return to work after stroke’, *South African Journal of Physiotherapy* 69(4), a378. 10.4102/sajp.v69i4.378

[CIT0057] Ojagbemi, A., Owolabi, M., Arulogun, O., Akinyemi, J., Akpa, O., Sarfo, F.S. et al., 2017a, ‘Prevalence and predictors of anxiety in an African sample of recent stroke survivors’, *Acta Neurologica Scandinavica* 136(6), 617–623. 10.1111/ane.12766 28417454 PMC5886726

[CIT0058] Ojagbemi, A., Akpa, O., Elugbadebo, F., Owolabi, M. & Ovbiagele, B., 2017b, ‘Depression after stroke in sub-Saharan Africa: A systematic review and meta-analysis’, *Behavioural Neurology* 2017, 4160259. 10.1155/2017/4160259 28819339 PMC5551463

[CIT0059] Palmcrantz, S., Holmqvist, L.W. & Sommerfeld, D.K., 2014, ‘Young individuals with stroke: A cross sectional study of long-term disability associated with self-rated global health’, *BMC Neurology* 14, 20. 10.1186/1471-2377-14-20 24472373 PMC3910684

[CIT0060] Pate, R.R., Pratt, M., Blair, S.N., Haskell, W.L., Macera, C.A., Bouchard, C. et al., 1995, ‘Physical activity and public health: A recommendation from the Centers for Disease Control and Prevention and the American College of Sports Medicine’, *JAMA* 273(5), 402–407. 10.1001/jama.1995.03520290054029 7823386

[CIT0061] Pickard, A.S., Johnson, J.A., Feeny, D.H., Shuaib, A., Carriere, K.C. & Nasser, A.M., 2004, ‘Agreement between patient and proxy assessments of health-related quality of life after stroke using the EQ-5D and Health Utilities Index’, *Stroke* 35(2), 607–612. 10.1161/01.STR.0000110984.91157.BD 14726549

[CIT0062] Raju, R.S., Sarma, P.S. & Pandian, J.D., 2010, ‘Psychosocial problems, quality of life, and functional independence among Indian stroke survivors’, *Stroke* 41(12), 2932–2937. 10.1161/STROKEAHA.110.596817 20966411

[CIT0063] Rankin, J., 1957, ‘Cerebral vascular accident in patients over the age of 60’, *Scottish Medical Journal* 2(5), 200–215. 10.1177/003693305700200504 13432835

[CIT0064] Rhoda, A., Smith, M., Putman, K. & Mpofu, R., 2014, ‘Motor and functional recovery after stroke: A comparison between rehabilitation settings in a developed versus a developing country’, *BMC Health Services Research* 14(82), 1–7. 10.1186/1472-6963-14-82 24559193 PMC3974037

[CIT0065] Rhoda, A.J., 2014, ‘Health- related quality of life of patients six months poststroke living in the Western Cape, South Africa’, *African Journal of Disability* 3(1), Art. #126, 6 pages. 10.4102/ajod.v3i1.126 PMC5443046 28730004

[CIT0066] Salter, K., Campbell, N., Richardson, M., Mehta, S., Jutai, J., Zettler, L. et al., 2013, ‘Outcome measures in stroke rehabilitation’, *Evidence-Based Review of Stroke Rehabilitation* 10(1), 1–144.

[CIT0067] Scheffler, E. & Mash, R., 2019, ‘Surviving a stroke in South Africa: Outcomes of home-based care in a low-resource rural setting’, *Top Stroke Rehabilitation* 26(6), 423–434. 10.1080/10749357.2019.1623473 31169468

[CIT0068] Sulla, V. & Zikhali, P., 2018, *Overcoming poverty and inequality in South Africa: An assessment of drivers, constraints and opportunities (English)*, World Bank, Washington, DC, viewed from http://documents.worldbank.org/curated/en/530481521735906534/Overcoming-Poverty-and-Inequality-in-South-Africa-An-Assessment-of-Drivers-Constraints-and-Opportunities.

[CIT0069] Tang, W.K., Lau, C.G., Mok, V., Ungvari, G.S. & Wong, K., 2013, ‘Impact of anxiety on health-related quality of life after stroke: A cross-sectional study’, *Archives of Physical Medicine and Rehabilitation* 94(12), 2535–2541. 10.1016/j.apmr.2013.07.012 23911556

[CIT0070] Taylor, A. & Ntusi, N.A.B., 2019, ‘Evolving concepts of stroke and stroke management in South Africa: *Quo vadis*?’, *South African Medical Journal* 9(2), 69–71. 10.7196/SAMJ.2019.v109i2.00009

[CIT0071] Unibaso-Markaida, I., Iraurgi, I., Ortiz-Marques, N. & Martinez-Rodrigues, S., 2019, ‘Degree of Functionality and perception of health-related quality of life in people with moderate stroke: Differences between ischemic and hemorrhagic typology’, *Behavioural Neurology* 2019, ID3405696. 10.1155/2019/3405696 PMC6512076 31182979

[CIT0072] Vestling, M., Tufvesson, B. & Iwarsson, S., 2003, ‘Indicators for return to work after stroke and the importance of work for subjective well-being and life satisfaction’, *Journal of Rehabilitation Medicine* 35(3), 127–131. 10.1080/16501970310010475 12809195

[CIT0073] Western Cape Rehabilitation Centre (WCRC), 2021, *Home page*, viewed 30 September 2021, from www.wcrc.co.za.

[CIT0074] Westerlind, E., Persson, H.C. & Sunnerhagen, K.S., 2017, ‘Return to work after stroke in working age persons; A six- year follow up’, *PLoS One* 12(1), e0169759. 10.137/journalpone.0169759 28061507 PMC5218734

[CIT0075] Widar, M., Samuelsson, L., Karlsson-Tivenius, S., Ahlstrom, G., 2002, ‘Long-term pain conditions after a stroke’, *Journal of Rehabilitation Medicine* 34(4), 165–170. 10.1080/16501970213237 12201611

[CIT0076] World Health Organization, 2011, *World report on disability*, WHO, Geneva.

[CIT0077] Yeoh, Y.S., Koh, G.C-H., Tan, C.S., Tu, T.M., Singh, R., Chang, H.M. et al., 2019, ‘Health-related quality of life loss associated with first-time stroke’, *PLoS One* 14(1), e0211493. 10.1371/journal.pone.0211493 30689666 PMC6349359

